# A User-Centric Context-Aware Framework for Real-Time Optimisation of Multimedia Data Privacy Protection, and Information Retention Within Multimodal AI Systems

**DOI:** 10.3390/s25196105

**Published:** 2025-10-03

**Authors:** Ndricim Topalli, Atta Badii

**Affiliations:** Department of Computer Science, University of Reading, Reading RG6 6AH, UK

**Keywords:** privacy engineering, soft biometrics, hard biometrics, re-identification, data intelligibility, multimodal data, privacy protection, context-aware AI, user-centric privacy, GDPR compliance, dynamic privacy adaptation, real-time data obfuscation

## Abstract

The increasing use of AI systems for face, object, action, scene, and emotion recognition raises significant privacy risks, particularly when processing Personally Identifiable Information (PII). Current privacy-preserving methods lack adaptability to users’ preferences and contextual requirements, and obfuscate user faces uniformly. This research proposes a user-centric, context-aware, and ontology-driven privacy protection framework that dynamically adjusts privacy decisions based on user-defined preferences, entity sensitivity, and contextual information. The framework integrates state-of-the-art recognition models for recognising faces, objects, scenes, actions, and emotions in real time on data acquired from vision sensors (e.g., cameras). Privacy decisions are directed by a contextual ontology based in Contextual Integrity theory, which classifies entities into private, semi-private, or public categories. Adaptive privacy levels are enforced through obfuscation techniques and a multi-level privacy model that supports user-defined red lines (e.g., “always hide logos”). The framework also proposes a Re-Identifiability Index (RII) using soft biometric features such as gait, hairstyle, clothing, skin tone, age, and gender, to mitigate identity leakage and to support fallback protection when face recognition fails. The experimental evaluation relied on sensor-captured datasets, which replicate real-world image sensors such as surveillance cameras. User studies confirmed that the framework was effective, with over 85.2% of participants rating the obfuscation operations as highly effective, and the other 14.8% stating that obfuscation was adequately effective. Amongst these, 71.4% considered the balance between privacy protection and usability very satisfactory and 28% found it satisfactory. GPU acceleration was deployed to enable real-time performance of these models by reducing frame processing time from 1200 ms (CPU) to 198 ms. This ontology-driven framework employs user-defined red lines, contextual reasoning, and dual metrics (RII/IVI) to dynamically balance privacy protection with scene intelligibility. Unlike current anonymisation methods, the framework provides a real-time, user-centric, and GDPR-compliant method that operationalises privacy-by-design while preserving scene intelligibility. These features make the framework appropriate to a variety of real-world applications including healthcare, surveillance, and social media.

## 1. Introduction

The wide adoption of multimodal recognition systems has occurred because of Artificial Intelligence (AI) and Machine Learning (ML) advancements that enable the capture and processing of multimedia data content, including face, object, emotion, scene, and action data for applications in various fields [1]. These technologies are deployed to automate processes, improve efficiency, and aid in decision making; however, they also present many privacy risks as they process vast amounts of Personally Identifiable Information (PII) such as facial biometrics, location data, and behavioural patterns. For example, sensor-based monitoring in workplaces or smart environments can unintentionally expose sensitive behaviours and social interactions [2]. As Sharma [3] highlights, privacy inconsistency causes users to engage in information disclosure behaviour despite their concerns about privacy risks, while privacy policies remain challenging to create because of this inconsistency [4].

To address these concerns, Privacy Engineering (PE) has emerged as a discipline focused on integrating privacy-by-design principles into AI systems as proposed by Martin and Alamo [5]. Hansen, Meiko Jensen and Martin Rost [6] define PE as a systematic approach to ensure adequate data protection within organisational systems. However, most current privacy protection AI systems rely on static obfuscation techniques, such as blurring, pixelation and masking, without dynamically adjusting to the scene contexts or user preferences [7,8,9,10,11,12,13,14,15]. Since these privacy challenges occur directly in data streams captured by vision sensors such as cameras, protecting sensor outputs becomes crucial to ensuring compliance and usability. The static obfuscation techniques lead to suboptimal privacy protection through over-masking data, thus reducing scene usability or under-masking data, which results in inadequate protection of sensitive data and failing GDPR compliance. The challenge is amplified in shared and dynamic settings, including smart homes, workplaces, or public events, where privacy preferences differ between users and contextual relationships among users and scene contexts [2,16]. Despite advances in current privacy protection methods, they still do not provide an integrated evaluation of multimodal recognition systems that combine soft-biometric traits, contextual reasoning, and user-defined privacy preferences in dynamic environments.

To overcome these limitations, we propose a user-centric, ontology-driven, and context-aware privacy protection framework that enables real-time and adaptive obfuscation. It uses semantic classification of recognised entities such as users, scenes, actions, objects, emotions, soft biometric traits, including gait, hair, clothing, as well as contextual privacy factors. This framework draws from Nissenbaum’s Contextual Integrity theory [17], which defines that privacy protection is not absolute but must be preserved to contextual norms such as “who is sharing what with whom and under what conditions”.

In this research, privacy context is defined as the combination of actors (users), their roles, actions, relationships, and the situational parameters that determine how data should be protected. This builds upon the ontology-based privacy protection models developed by Badii, Tiemann, and Thiemert [13], where ontologies encode relationships between entities, actions, contexts, and privacy rules to enable privacy decision reasoning. Environment, in this context, refers to the spatial, temporal, and interactional conditions in which data is captured and shared. By reasoning over these elements, the framework dynamically interprets privacy context and decides if data should be obfuscated [13].

The proposed framework introduces several key innovations:

- The use of soft biometric traits such as gait, hair type, hair colour, skin tone, age, and gender for fallback re-identification when face recognition fails to detect and recognise individuals because of occlusions, addressing the limitations of facial masking alone [18,19,20].
- A Re-Identifiability Index (RII) that computes the likelihood of identifying a user based on soft biometric features, enabling a detailed privacy control.
- An Auto Privacy mode that uses machine learning to predict privacy preferences based on contextual data and historical behaviour, to improve on current methods that cannot adapt to dynamic environments.
- Support for user-defined red lines, such as “always hide logos”, that override any other privacy settings, which ensures that user priorities are always enforced.
- A rule-based ontology model that defines the relationships between users, entities, contexts, and privacy levels for consistent and explainable privacy decisions.

Prior studies validate the importance of this framework. Lin and Li [20] show that using 23 out of 30 soft attributes can yield 85% re-identification accuracy, and that combining soft traits such as hair, gender, and age boosts recognition performance by up to 6%. Similarly, Bari and Gavrilova [18] and Corbishley, Nixon, and Carter [19] report re-identification rates of 85% when using gait and other soft biometrics, which highlight the limitations of facial masking alone.

The proposed framework builds upon these gaps by treating privacy as a multidimensional and context-sensitive process, which applies real-time obfuscation based on scene context and user-defined privacy settings. The multimodal AI pipeline of the framework integrates the YOLOv5 model for object recognition, MTCNN for face recognition, SlowFast for action recognition, Places365 for scene classification, and EfficientNet for emotion recognition. The recognition outputs are used to recognise individual user, identify privacy contexts and inform the ontology-driven privacy engine to dynamically apply obfuscations while preserving scene intelligibility and GDPR compliance. The framework also supports user-centric privacy in shared spaces by encoding privacy rules into an ontological model that ensures transparency, scalability, and explainability.

## 2. Related Work

Given the expansion of multimodal AI applications, concerns regarding personal privacy have increased, specifically in processing PII within video data. Although previous research [7,8,9] addresses privacy-preserving techniques, significant gaps remain in such approaches, as many rely on static obfuscation rules and do not adapt to user-defined preferences, real-time contextual shifts, or multi-user scenarios. As a result, these models either over-mask content, undermining usability, or under-mask sensitive data, compromising privacy and GDPR compliance.

Recent efforts in privacy protection have explored soft biometrics as both a challenge and an opportunity. Zhou, Pun, and Tong [14] highlight the limited exploration of dynamic face pixelation as a method and its inefficiencies in highly dynamic settings. Similarly, Hasan, Shaffer, Crandall, and Kapadia [9] and Lin and Li [20] demonstrate that soft biometric features, such as gait, hair-type, skin tone, age, and clothing attributes can lead to re-identification even when faces are obscured. For instance, Lin and Li [20] show that using 23 out of 30 soft attributes can yield an 85% identification rate, reinforcing the privacy risks posed by non-facial attributes. However, few systems integrate these cues into a coherent privacy enforcement model.

Existing privacy protection methods either do not recognise soft biometric features to identify individuals with the aim of personalised privacy protection or fail to dynamically adjust obfuscation based on contexts or user red line. In contrast, the proposed framework improves on this by incorporating a user-centric, ontology-driven privacy framework that models the relationships between users, visual entities (faces, objects, etc.), environmental context, and user-defined privacy red lines. This framework incorporates the following:

- Soft biometric analysis as both a fallback to face recognition and a standalone re-identifiability risk factor.
- A Re-Identifiability Index (RII) that quantifies re-identifiability risk and advises dynamic masking decisions.
- Support for user-defined red lines, such as “always hide logos”, which override any predefined settings.
- Support for Auto Privacy through supervised learning, predicting privacy settings needs based on scene type, emotional state, and prior user behaviour.
- An ontology-based reasoning model that defines the relationships between users, entities, contexts, and privacy levels for consistent privacy decision-making.

Unlike prior methods such as those of Hasan, Shaffer, Crandall and Kapadia [9] and Zhou, Pun and Tong [14], which apply uniform, static rules, the proposed framework uses semantic inference to guide privacy decisions on a frame-level and user-centric basis. It addresses the balance between intelligibility and privacy by using contextual cues such as location, scene category, or action type, and balancing these with user-defined privacy settings and red lines.

The proposed framework extends the state of the art by embedding contextual integrity, real-time adaptability and re-identifiability assessment within a unified and scalable privacy protection pipeline. Its ontology-based reasoning capability enables the framework to reason over context, which makes it particularly effective in complex and multi-user environments. In doing so, it directly addresses key challenges in intelligent, context-aware privacy preservation.

### 2.1. Privacy Challenges and User-Centric Risks in Multimodal AI Systems

Modern AI systems increasingly combine multiple recognition capabilities, including face, scene, object, emotion, and action recognition, to enable more automatic operations within a variety of applications. However, the processing of such large quantities of PII data creates major privacy risks while posing challenges to data security, user control, and regulatory standards specifically outlined under GDPR [21]. The ability of AI systems to extract specific attributes such as identity and location data points leads to serious privacy issues regarding profiling practices, mass surveillance, and unauthorised data misuse [22].

The continuous growth of location-based services intensifies this concern as stated by Jiang, Li, Zhao and Zeng [2], that the ubiquity of GPS-enabled applications has led to pervasive location tracking. Castillo [23] reveals that 94% of smartphone users conduct searches for location-specific data, and 72% are targeted by location-aware advertisements, which indicates the comprehensive utilisation of personal data for both commercial and possibly intrusive activities.

A key challenge is the lack of adaptive privacy methods as existing privacy methods use static privacy models and fail to adapt to the changing user preferences, entity sensitivity, and dynamic contexts [7,9,16]. These methods use anonymisation techniques such as blurring and pixelation that provide a level of privacy protection [8]. However, they fail to match diverse user requirements and provide inadequate or excessive privacy protection to users [22], and they seriously diminish data utility as shown by Hasan, Shaffer, Crandall, and Kapadia [9]. Insufficient protection could lead to re-identifying anonymised data through cross-referencing with external datasets, making privacy protection countermeasures complex and difficult to manage as an evolving requirement [24]. More critically, re-identification through soft biometric traits, like gait, hair colour, age, or clothing style, can be used to cross-reference and identify individuals even after standard anonymisation. Sosa, Fierrez, and Vera-Rodriguez [25] demonstrate that using a wide range of soft biometric attributes can yield re-identification accuracies exceeding 85%, raising significant risks that most systems fail to address.

Another major challenge for organisations today is regulatory compliance. The GDPR requires necessary data minimisation tactics alongside transparency about data use and formal consent (European Commission, 2016). However, most AI systems fail to effectively implement privacy-by-design solutions that are transparent, customisable, and adaptable based on individual preferences according to Gurses, Troncoso and Diaz [26]. Current permission-based frameworks show inadequate results because users do not understand them well enough and lack the ability to adapt to different contexts [22]. Additionally, current methods are not adequately developed to effectively manage multi-user privacy requirements noted by Sezer, Dogdu, and Ozbayoglu [27]. The collaborative AI environments within smart homes and video conferences require individual and robust privacy settings regardless of differing user requirements, as noted by Ren, Lee, and Ryoo [10]. The predefined privacy options used in current systems fail to adapt dynamically to changing contexts, user states, or detected objects, leading to privacy vulnerabilities.

### 2.2. Context-Aware and Multi-User Privacy Adaptation Techniques

Privacy management within multi-user settings presents specific challenges in video conferencing, surveillance, and collaborative areas, according to Sezer, Dogdu, and Ozbayoglu [27]. Guo, Zhang, Hu, He, and Gao [28] highlight that current privacy settings typically apply uniform privacy configurations across all users and disregard individual preferences and contextual interactions. This results in two key limitations: over-protection, where excessive obfuscation reduces scene intelligibility and usability, or under-protection, where privacy-sensitive user data remains insufficiently protected [29].

A major limitation in existing approaches is their lack of adaptability to dynamic group interactions and contexts. Gurses, Troncoso, and Diaz [26] highlight the need for real-time, personalised privacy control, that could ensure that each user’s privacy settings are maintained while enabling seamless collaboration. Most current systems do not support context recognition or negotiation between conflicting user preferences.

To address this, our framework adopts a context-aware and ontology-driven design, dynamically adjusting privacy protection [10] according to scene type, recognised entities, user interactions, and individual privacy preferences. It builds upon Nissenbaum’s Contextual Integrity theory [17], which conceptualises privacy as a context-bound expectation based on appropriate information flow between actors, under specific roles and transmission principles. Privacy is considered violated when personal data is shared outside of these context-appropriate boundaries, for example, when a bedroom scene is shared publicly without user consent.

This work extends and builds on the foundational contributions by Badii, Al-Obaidi, Einig and Ducournau [11], who introduced the Holistic Privacy Impact Assessment (H-PIA) framework, and by Badii and Al-Obaidi [12], who demonstrated privacy protection via semantic scene classification. We extend these ideas with real-time multimedia analysis and user-defined rules, delivering an explainable and scalable framework. Furthermore, Badii, Tiemann, and Thiemert [13] highlight that privacy reasoning should integrate heterogeneous data sources into a unified model.

### 2.3. Performance vs. Privacy Trade-Offs in AI Systems

Balancing privacy protection and performance is a major challenge in multimodal AI systems especially under real-time conditions. Studies confirm that privacy-preserving techniques help protect against re-identification according to Narayanan and Shmatikov [24], yet Liu, Song, Liu, and Zhang [29] demonstrated that privacy-enhancing mechanisms may lead to accuracy degradation, creating trade-offs between usability and privacy robustness. Real-time AI applications should have a balance between computational efficiency and privacy protection, as these systems require high-speed processing. Studies by Sezer, Dogdu, and Ozbayoglu [27] and Zhou, Wang, Liang, and Wang [30] emphasise that sophisticated privacy methods such as obfuscation and encryption produce latency and require high computational resources. Gurses, Troncoso, and Diaz [26] further underscore the need for efficiency in resource-constrained environments, as excessive computational load can hinder responsiveness and user experience.

Privacy protection through obfuscation measures helps protect sensitive data but can lead to performance reductions and visual interpretability. The findings from Olejnik, Dacosta, Machado, and Huguenin [22] demonstrate that over obfuscation reduces system reliability by causing performance problems between maintaining privacy integrity and preserving scene intelligibility.

### 2.4. Comparison of Existing Approaches and Research Gaps

Existing privacy-preserving approaches in multimodal AI systems fall into static privacy models and permission-based frameworks, both of which face significant limitations in handling dynamic user preferences, contextual variability, and multi-user interactions. The current privacy-preserving approaches encounter significant limitations when dealing with critical real-time AI applications that process large volumes of PII, such as facial attributes, emotional expressions, and behavioural cues.

Conventional anonymisation techniques, such as blurring and pixelation, provide a fixed level of privacy protection as stated by Frome, Cheung, and Abdulkader [8], but fail to accommodate evolving user needs or varying sensitivity levels of data stated by Hassan, Shaffer, Crandall, and Kapadia [9]. Narayanan and Shmatikov [24] demonstrate that static obfuscation methods may fail to prevent re-identification when combined with external data, hence rendering privacy protections less effective in real-world applications. Similarly, Olejnik, Dacosta, Machado, and Huguenin [22] state that systems that rely on manual user settings meet usability challenges, as users often struggle to understand and manage their privacy settings effectively. Additionally, Gurses, Troncoso, and Diaz [26] and Ren, Lee, and Ryoo [10] have shown that current AI-driven privacy methods lack transparency, and context-awareness and do not scale well in real-time, multi-user or real-time environments.

Moreover, recent studies, such as Sezer, Dogdu, and Ozbayoglu [27] report that current privacy methods use fixed general privacy settings that fall short of accommodating modern AI-driven applications, including social media platforms and collaborative workspaces. Liu, Song, Liu, and Zhang [29] further identify key challenges in ensuring real-time efficiency, scalability, and GDPR compliance. They pointed out that existing privacy mechanisms often result in over-protection, which reduces data usability, or under-protection, which compromises privacy protection.

Among the few context-aware frameworks, the Holistic Privacy Impact Assessment (H-PIA) framework by Badii, Al-Obaidi, Einig, and Ducournau [11] represents a significant contribution. Their model treats privacy filtering as a multi-layered process involving technical and human-centric factors.

Later work by Badii and Al-Obaidi [12] introduced a context-aware filtering strategy that applied different obfuscation techniques to face, skin, and body regions. Their framework aimed to balance privacy, intelligibility, and pleasantness, taking under consideration recognisable attributes such as race and gender still impacted perceived privacy. Although this marked progress toward adaptive privacy filtering, it lacked semantic reasoning, user-defined red lines, or integration with multimodal entity recognition at the data-instance level (e.g., recognising faces, objects, and actions within each frame and assigning them context-specific re-identifiability risks).

Further foundational work by Badii, Tiemann, and Thiemert [13] proposed the use of semantic data integration and ontology-based modelling for improving situational awareness in security applications. Their system showed that data from heterogeneous sources, such as CCTV footage, could be unified under an ontology-driven structure that enabled rule-based reasoning and decision support. While not directly focused on user-centric privacy, their methodology forms a critical foundation for semantic reasoning and context modelling adopted in the proposed framework.

Building upon these foundational works, this research proposes a real-time, user-centric, and ontology-driven privacy protection framework that operationalises contextual reasoning through entity-level sensitivity classification, soft-biometric risk modelling, and adaptive obfuscation. It unifies the technical robustness of earlier privacy filters, the context-aware aspirations of MediaEval [12] methods, and the structured semantic reasoning of MOSAIC [13] under a scalable, GDPR-compliant, and multi-user capable system for privacy protection in multimodal AI.

## 3. Methodology

### 3.1. Framework Overview

The proposed user-centric, context-aware privacy protection framework integrates multimodal AI recognition with adaptive enforcement to protect PII in real time. All recognition and privacy enforcement operate on data captured by vision sensors (cameras). Detected elements are classified based on sensitivity, and privacy levels are dynamically updated based on user-defined preferences, contextual factors, soft-biometric attributes, and the Re-Identifiability Index (RII) that quantifies re-identification risk. The framework is structured into three main modules, where each is responsible for a specific aspect of privacy adaptation and enforcement:

1. The Recognition modules use state-of-the-art AI models such as YOLOv5 for object recognition, MTCNN for face recognition, and Places365 for scene classification to extract and analyse contextual information from video streams. It identifies privacy-sensitive entities, including faces, objects, actions, emotions, and scenes, along with soft biometrics, including gait, hair type, age, and gender, that are used to calculate the RII, when facial recognition fails. These recognition outputs are passed into contextual risk analysis for real-time privacy decision-making.
2. The Privacy Enforcement module computes the appropriate privacy levels dynamically by classifying detected entities into privacy-sensitive categories such as private, semi-private, or public. Based on sensitivity, it applies privacy-preserving techniques such as blurring, pixelation, silhouette masking, or synthetic data replacement (GAN-based anonymisation) [31]. The framework aligns obfuscation intensity with user preferences and entity sensitivity to maintain a balance between privacy protection and usability. In multi-user settings, it supports personalised enforcement, strictly protecting high-privacy users even when others share lower privacy levels.
3. Privacy Reasoning and User Context Module captures and reasons over user-defined privacy rules using ontology-based logic to ensure structured, consistent, and context-aware decision-making. It supports multiple privacy modes (Auto Privacy, High, Medium, Low, and No Privacy), enforces red-line rules (e.g., always hide logos), and adapts protections in real time based on scene dynamics and feedback from AI recognition modules. It also handles Auto Privacy mode, which uses supervised learning to predict preferred privacy configurations from historical user behaviour and contextual cues.

**Figure 1:**
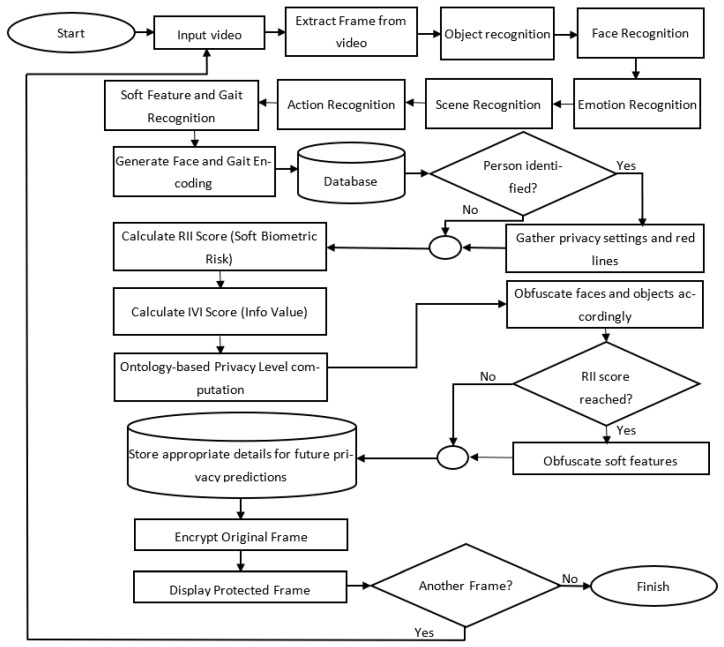
Architecture diagram showing flow from recognition to ontology-driven privacy adaptation.

By combining multimodal recognition, dynamic privacy adaptation, ontology-based reasoning, and user-driven privacy settings, this framework maintains strong privacy protection without compromising scene intelligibility. It is deployable in a variety of environments, including smart homes, video conferencing platforms, and public surveillance contexts. Figure 1 illustrates the high-level architecture, showing the recognition modules, context reasoning, and enforcement pipelines that deliver adaptive privacy protection.

This layered design supports real-time, context-aware privacy decisions by integrating multimodal recognition, ontology-based reasoning, and user-centric policy enforcement into a unified, adaptive framework.

### 3.2. Context-Sensitive Privacy Mechanisms

Context-sensitive privacy ensures that privacy protection is dynamically adjusted based on the sensitivity of detected entities and environmental context. Unlike static privacy models [7,8,9], which apply fixed privacy settings regardless of context, our framework follows Nissenbaum’s Contextual Integrity Theory [17], which asserts that privacy norms depend on the interplay between actors, information types, and contextual settings. To operationalise this, the framework combines ontology-driven knowledge representation with real-time AI inference. The ontology captures privacy preferences alongside contextual semantics such as scene type, emotional expression, action, and soft biometric traits. It enables reasoning over privacy decisions by evaluating what is shown, to whom, in what context, and under what user-defined constraints. We define two types of contexts that shape privacy decisions:

1. Frame context, which defines what is happening on the scene (e.g., people, objects).
2. Exposure, where and to whom the context will be visible (e.g., social media, shared in public, private message).

These contexts influence both the user’s expressed privacy preferences and the adaptive privacy reasoning of the framework. For instance, being at home with friends (private frame context) may trigger different masking behaviour than being in a public park (public frame context), especially if the intended exposure is social media. Such differences are modelled by the ontology to balance privacy risks and intelligibility across platforms.

Previous studies show that soft biometric features offer significant potential for user re-identification. Bari and Gavrilova [18] state that users can be identified using gait biometrics with a 98.08% accuracy. Corbishley, Nixon, and Carter [19] identified that combining soft biometric features can increase the re-identification accuracy up to 88.1%, depending on the soft biometric features and the combinations used. Moctezuma, Conde, Diego, and Cabello [32] introduce a person identification method using only three soft biometrics features, such as clothing, complexion, and height, to reach an 85% identification rate.

Additionally, the study explores how a recognition system using soft biometric features such as gender, backpack, jeans, and short hair achieves 53–75% accuracy. This aligns with the findings by Sosa, Fierrez, and Vera-Rodriguez [25], who demonstrate that using a broader set of 73 soft biometrics can further improve re-identification accuracy, reaching 85.54%. Expanding on this, Corbishley, Nixon, and Carter [19] identified that combining key features such as gender, height, skin tone, hair colour, hair type, age, and so on can result in re-identification of individuals and quantification of soft features (see Table 1). For this work, only the soft features with the highest weights are selected to improve the re-identifiability.

To support re-identification when face recognition is inconclusive, these soft biometric traits are analysed to compute a Re-Identifiability Index (RII) to determine privacy enforcement. If the RII exceeds a threshold, the framework associates the user with stored privacy settings or increases obfuscation to mitigate re-identification risk. This adaptive mechanism aligns with Contextual Integrity by updating privacy to contextual norms rather than static rules, ensuring that privacy decisions are context-aware and personalised.

Ontology-driven privacy enforcement rules follow a hierarchical sensitivity model, where the highest-sensitivity element detected (e.g., scene, object, action, and emotion) in a frame determines the final privacy level applied. Additionally, user-defined red lines, such as specific features, objects, logos, or individuals that must always be masked, are enforced independently of contextual sensitivity, ensuring that user-specified constraints override general framework predictions when necessary. Users on Auto Privacy mode benefit from the supervised learning model that dynamically predicts and applies optimal privacy settings based on historical and contextual cues. The resulting privacy enforcement mechanism combines hierarchical sensitivity reasoning with the user-defined red lines, ensuring both adaptive flexibility and strict user control. Table 2 summarises the privacy actions applied under different user settings and contextual conditions.

This adaptive, user-centric, and explainable model enables privacy enforcement that is personalised and scalable. It addresses long-standing gaps in privacy mechanisms, as noted by Halvatzaras and Williams [33] and Laak, Litjens, and Ciompi [34], who emphasise the importance of adaptable privacy models that respond to changing user and environmental contexts. It also addresses concerns raised by Olejnik, Dacosta, Machado, and Huguenin [22] regarding the lack of effective privacy mechanisms in AI systems.

### 3.3. Multi-User Privacy Protection

Current privacy models produce ineffective results by neglecting dynamic privacy requirements between multiple users who share video streams, use smart homes, and in public surveillance systems, where multiple individuals may have diverse privacy preferences. Research by Ren, Lee, and Ryoo [10] highlights that current privacy systems enforce static privacy configurations for all users without considering individual privacy requirements. Similarly, Olejnik, Dacosta, Machado, and Huguenin [22], and Sezer, Dogdu, and Ozbayoglu [27], identified a lack of adaptive mechanisms that prioritise privacy-sensitive users, contextual sensitivity, and usability considerations.

To address these gaps, the proposed framework integrates real-time, multi-user, privacy enforcement that dynamically adjusts privacy settings for each detected user. It evaluates three primary factors: user-defined privacy preferences (e.g., Auto Privacy, High Privacy, Medium Privacy, Low Privacy, or No Privacy), contextual sensitivity of the detected entities (objects, actions, and emotions), and the presence of soft biometric traits that may lead to re-identification. When multiple users appear on the same frame, the framework prioritises the highest privacy level for shared scene elements, while still applying individualised obfuscation to each person. For example, if User A opts for High Privacy while User B selects No Privacy, shared sensitive objects will all be obfuscated, but User B’s face will remain unobscured, which ensures a balance between collective protection and personal choice.

The ontology-driven rule engine resolves conflicts between users using a hierarchical model and user-specific red lines, such as “always hide logos”, that override contextual conflicts. Additionally, when a user is not directly recognised (e.g., face is occluded), the framework applies fallback privacy prediction based on soft biometric features and the Re-Identifiability Index (RII). In Auto Privacy mode, privacy settings are predicted using a supervised model trained on user historical data and contextual cues, which ensures users remain protected even when identity is ambiguous.

This user-centric, multi-user framework ensures adaptive privacy protection, and it maintains compliance with GDPR and user-defined privacy constraints while preserving scene intelligibility and usability in complex settings.

### 3.4. Person Re-Identification

In scenarios where facial recognition is not possible, due to occlusion, low resolution, or user-defined masking, soft biometric traits are used as an alternative means for user re-identification and RII calculation. These traits include gait, hair type, skin tone, age, gender, and clothing/accessory cues, all of which provide varying degrees of identifiability. As Dantcheva, Elia, and Ross [35] highlight, a single soft biometric trait would not be unique enough to identify a subject, but their combination can significantly increase the probability of identity inference [32,36]. Dantcheva, Elia, and Ross [35] further discuss that every soft feature can carry information about different soft biometric traits; for instance, hair type may implicitly indicate ethnicity or gender. To systematically evaluate the risk of re-identification, Dantcheva, Velardo, and Dugelay [36] propose categorising each soft biometric feature into a distinctiveness level of Low, Medium, or High.

To address re-identifiability risks when using soft features, the proposed framework integrates two key components:

- Re-Identifiability Index (RII) score that quantifies the cumulative re-identification risk of a user based on identified soft biometric features.
- Intelligibility Value Index (IVI) measures the balance between obfuscation and information value of a given trait within the current frame and context.

IVI is not yet a formally standardised metric in the existing literature; however, it draws inspiration from several foundational works. Moctezuma, Conde, Diego, and Cabello [32] introduce a numbering points system for the list of features to calculate feature weights, while Bari and Gavrilova [18] and Corbishley, Nixon, and Carter [19] quantified main soft biometric features based on their contribution to re-identification likelihood. Building upon these models, the proposed framework introduces the Intelligibility Value Index (IVI), which measures the proportion of scene interpretability retained after obfuscation of high-RII features and calculated as shown in Equation (1).

(1) $$IVI\;Score=\frac{Retained\;Interpretability\;Weight}{Total\;Interpretability\;Weight}$$

A higher IVI indicates that obfuscation has minimally impacted the ability to interpret the scene, whereas a lower IVI signals substantial loss of semantic content. Importantly, IVI is evaluative and does not drive obfuscation decisions directly but provides a quantitative measure of the framework effectiveness in preserving scene intelligibility. The results of this evaluation are summarised in Table 3.

When face recognition fails, the framework activates fallback matching using soft biometric embeddings (gait, body shape, hair, and clothing). If a user profile is identified, their privacy settings and red lines are used for obfuscation. If no match exists, RII scores are used to identify potential re-identification risk and enforce protective masking as needed.

This layered framework supports privacy continuity in real-time applications by dynamically balancing privacy protection with scene intelligibility and in alignment with Nissenbaum’s Contextual Integrity theory [17].

### 3.5. Privacy Risks and Mitigation

The increasing accuracy of soft biometric-based re-identification presents significant privacy challenges in multi-user, real-time video processing systems. As demonstrated in prior research [18,25], soft features such as gait, skin tone, hair type, and clothing can be used to identify individuals even when facial data is obscured. This raises concerns in shared settings, where individuals may not directly interact with the system, but their data is collected without consent.

Soft biometric features, including gait patterns (with an accuracy up to 98.08% [18]) or a combination of gender, hair type, skin tone, clothes, and others (with an accuracy up to 88.1% [19]), could enable re-identification of individuals. In scenarios where face recognition fails, these features may still facilitate tracking, which violates user anonymity. This risk is increased in social media or surveillance contexts, where exposure is less controlled.

To mitigate these risks, the proposed framework applies context-sensitive and user-centric privacy masking that dynamically adapts to user privacy settings. These include

- High-Risk Features such as soft biometric traits (e.g., gait, hair colour) are obfuscated in cases when the Re-Identifiability Index (RII) exceeds a defined threshold.
- Hierarchical sensitivity enforcement to prioritise the most sensitive element in the frame, scene, object, action, or emotion, and applies the strictest privacy level.
- Independently of the context or prediction, user-defined red lines (e.g., tattoos, logos, or specific clothing items) are always obfuscated.
- In shared settings, the proposed framework uses multi-user conflict resolution to identify the highest applicable privacy preference across users, which ensures that no individual’s privacy is compromised due to the lower preference of others.

The proposed framework complies with GDPR principles of data minimisation, privacy-by-design by default, and security of processing, as only essential data is processed and sensitive elements are masked by default. Adaptive masking further protects indirect identifiers (e.g., clothing) when re-identification risks are detected.

By combining contextual reasoning, soft biometric risk scoring, and user-centric privacy settings and red lines, the proposed framework provides robust protection against re-identification, even in complex or multi-user scenarios. This ensures that the privacy of individuals is protected, while preserving scene intelligibility.

### 3.6. Implementation Details

The proposed framework incorporates a modular architecture and ontology-driven reasoning to achieve dynamic, real-time, user-centric privacy protection across multimodal recognition tasks. It integrates state-of-the-art deep learning models for face, object, scene, action, and emotion recognition, operating in parallel to extract semantic features from continuous streams captured by vision sensors (cameras). These features are fed into a unified reasoning engine that evaluates user-defined red lines, contextual sensitivity, and re-identifiability risk to determine the appropriate level of privacy protection per frame. The framework is optimised for GPU-accelerated processing and is designed to prioritise real-time performance, GDPR compliance, and intelligibility preservation.

Multiple recognition models were evaluated based on accuracy and computational efficiency to ensure robust and real-time performance, with detailed results summarised in Appendix A (Table A1, Table A2, Table A3, Table A4 and Table A5). Among the evaluated models, MTCNN was selected for its balance between processing speed of 16–99 FPS, recognition accuracy of 94.4% and its ability to run on the GPU. AlexNet was selected for scene recognition as it achieved the best balance between classification accuracy of approximately 85% and computational efficiency of up to 205 FPS. YOLOv5 was selected for object recognition based on recognition speed of approximately 140 FPS and sufficient accuracy, and SlowFast Networks for action recognition for their ability to capture both detailed and rapid movements. The EfficientNet model was selected for emotion recognition because of its high processing speed of up to 155 FPS and competitive accuracy of 84.6%. Across all modules, model selection prioritised a balance between recognition precision and computational speed, ensuring real-time operation using GPU-accelerated hardware.

Privacy enforcement mechanisms automatically classify recognised entities using established privacy categories such as private, semi-private, and public. The framework ensures that the highest level of privacy settings is applied to each frame, maximising the protection of sensitive objects. To balance privacy protection with usability, the framework dynamically adjusts privacy settings based on the user predefined privacy setting, contextual classification of detected entities, and user-predefined red lines. In the cases where users are on Auto Privacy, the framework uses a machine learning model to continuously refine privacy recommendations based on past user interactions, scene attributes, and sensitivity levels. Soft biometric traits such as gait, clothing types and colours, clothing logos, hair type and colour, skin tone, gender, and age are further analysed to compute a Re-Identifiability Index (RII) for each user (described in Section 3.4), guiding dynamic soft feature masking decisions to further enhance user privacy where needed.

To support this process, the framework uses a dedicated database that stores recognised entities, detected from sensor inputs (video frames), including face encodings, objects, scenes, actions, emotions, and their corresponding sensitivity levels. This structured database functions as an important reference point for the recognition modules, enabling real-time, context-driven privacy adjustments based on live feedback. The reasoning engine integrates multimodal recognition, soft biometric scoring, and ontology-based context rules to balance privacy protection with scene intelligibility. Adaptive obfuscation is guided by RII and IVI scores, while user-defined red lines (e.g., “always hide logos”) override both contextual inference and risk scoring.

The framework is implemented using PyTorch version 2.9.0 and TensorFlow version 2.10.1 with CUDA version 11.8 optimisation, enabling deployment on devices with limited resources. The framework is designed to scale effectively across various real-world applications to maintain high privacy protection without hindering usability. A detailed comparison between the proposed framework and prior static, semi-dynamic, and context-aware methods (including Atta [11,12,13]). This demonstrates the significant improvements achieved in adaptability, contextual reasoning, soft biometric protection, and real-time performance.

### 3.7. Experimental Setup and Evaluation Metrics

A series of tests was performed on the proposed privacy protection framework by utilising sensor-captured datasets for each module (faces, objects, scenes, emotions, and actions), and each class label was categorised into privacy-sensitive categories. The framework processed video streams captured by web cameras to evaluate its ability to adapt to different conditions, such as different user requirements and privacy preferences.

The experimental setup included an NVIDIA RTX 4050 GPU, Intel i7-13620H CPU, and 32 GB RAM, providing sufficient computational power for real-time processing. The framework was developed using Python version 3.10.9, PyTorch version 2.9.0, TensorFlow version 2.10.1, and OpenCV version 4.7.0, using their GPU-accelerated capabilities and optimised execution pipelines to ensure efficient real-time processing for face, object, scene, action, and emotion recognition.

To ensure clarity of our experiments, the proposed framework parameters and model configurations are reported in Table 4. These parameters govern the recognition modules, privacy thresholds, and obfuscation methods used during evaluation. To evaluate the recognition modules, we used publicly available datasets (VGGFace2 [37], COCO [38], Places365 [39], Kinetics [40], and FER2013 [41]) for core recognition modules and bespoke datasets adapted for soft biometric features such as clothing styles and logos. To benchmark datasets, standard training/testing splits were followed, whereas for bespoke datasets, an 80/20 split was applied. Modules were benchmarked to balance recognition accuracy and computational efficiency for real-time processing, such as using a 0.7 confidence threshold for MTCNN and a 0.25 threshold for the YOLOv5s model.

To evaluate the adaptability of the privacy protection mechanisms, the framework was evaluated under various scenarios, including single-user and multi-user settings and variations in sensitivity, and different privacy levels, such as No Privacy, Low, Medium, High, or Auto Privacy. Recognition modules were evaluated to balance accuracy with computational efficiency and ensure feasibility in real-time operation.

In addition to recognition performance, the framework was assessed for its ability to balance privacy protection with scene intelligibility. Table 5 summarises the trade-offs between usability and re-identifiability risk across various features.

To evaluate how well the proposed framework manages this balance in real-time conditions, quantitative and qualitative evaluation methods were used. Quantitatively, the RII was used to assess privacy risks associated with identified soft biometric features, and IVI was used to capture information value and scene intelligibility. Computational latency and obfuscation effectiveness were also measured to ensure real-time feasibility. Qualitative evaluation involved structured user studies to assess participant’s perceived privacy protection, scene intelligibility after obfuscation, and overall usability.

Following prior work by Hasan, Shaffer, Crandall, and Kapadia [9], re-identification rates were adopted as a comparative benchmark to assess how well the framework prevents unintended identification. To ensure that privacy enforcement does not compromise usability, system performance was measured before and after privacy-preserving transformations were applied. Additionally, the evaluation included measuring the processing latency of the recognised entities, privacy settings, and necessary obfuscations for each frame to guarantee that privacy control remained effective and scalable in real-time.

Overall, the experimental setup provides a complete assessment of privacy enforcement techniques by maintaining a balance between privacy protection, recognition accuracy, computational performance, and user experience. The results highlight the balance involved in dynamic and context-aware privacy adaptation and demonstrate that the framework operates effectively on video streams in real-world applications.

### 3.8. Ethical Considerations

The proposed framework complies with ethical principles as it prioritises user privacy, transparency, fairness, and regulatory frameworks such as GDPR. Ensuring that privacy protection mechanisms align with legal and ethical standards is critical for responsible AI [43] deployment. A key ethical consideration is user consent and control so that individuals can exercise their decision-making power regarding privacy preferences. Individuals are provided with granular control over their privacy settings, enabling them to adjust their privacy configurations at any time based on personal comfort and identified contexts. This user-centric framework aligns GDPR standards of empowering users and gaining their consent, which helps create better user trust and ensure that privacy management remains transparent, explainable, and in the user’s hands.

The framework also incorporates data minimisation by processing only what is required for privacy protection and avoids unnecessary collection of personal information. The Re-Identifiability Index (RII), which uses soft biometric features such as gait, hair type, clothing, and skin tone, is used to quantify re-identification risk and to authenticate users when face recognition fails. This is effective but raises ethical concerns around transparency, profiling, and potential discrimination. To mitigate such risks, users are informed about how soft biometrics are used and may opt out of their inclusion at any time, ensuring compliance with fairness and consent principles.

Addressing fairness and bias mitigation is essential in multimodal AI privacy systems. Recognition models used to detect faces, objects, scenes, actions, and emotion information, along with the datasets they are trained on, are evaluated to identify potential demographic bias. The training data is carefully selected and, when necessary, is updated to ensure a balanced representation across demographic groups, reduce bias in privacy protection mechanisms, and operate equitably across all types of user groups.

Ethical design is a main principle of the proposed framework as it ensures a balanced integration of privacy protection, fairness, trust, and regulatory compliance. To protect sensitive data captured from video streams, the framework uses encryption protocols that encrypt raw data and store it in an encrypted format. Access to the encrypted data is restricted to authorised users only or government representatives. Moreover, data stored on the database is restricted through strict access control mechanisms, where each user has access only to their personal data.

This framework prevents unauthorised use or data exposure and is aligned with the GDPR data protection requirements. By integrating these ethical enforcements into its architecture, the framework represents a responsible, secure, and user-centric model to develop multimodal AI privacy protection.

## 4. Results and Analysis

This section presents a comprehensive evaluation of the proposed ontology-driven, user-centric privacy protection framework. The analysis draws on both quantitative and qualitative methods to assess its effectiveness across multiple dimensions, including privacy protection strength, visual intelligibility, computational performance, and user satisfaction. Each subsection examines key outcomes from experiments and user studies conducted in diverse, real-world and simulated environments involving varying user types, scene contexts, and data sensitivities, all based on data captured by vision sensors.

The evaluation framework is designed to test how well it balances privacy preservation with usability, particularly under dynamic and multi-user conditions. Central to this assessment are two core metrics developed in this work: the Re-Identifiability Index (RII), which estimates the risk of identifying individuals based on soft biometric traits, and the Intelligibility Value Index (IVI), which approximates how much semantic clarity is retained post-obfuscation. These metrics, alongside recognition accuracy, responsiveness, and subjective user feedback, form the basis for determining the real-world applicability of the proposed framework.

### 4.1. Intelligibility vs. Re-Identifiability

Balancing scene intelligibility with privacy protection is a main challenge in privacy-preserving multimedia systems. The proposed framework addresses this challenge by combining the RII, which quantifies the likelihood of user re-identification, with the IVI, which measures how much semantic content remains interpretable after obfuscation. These metrics are used to manage adaptive privacy decisions that respond to contextual risk and usability needs. The evaluation demonstrates that while increased obfuscation improves privacy, it may compromise intelligibility, which highlights the need for intelligent trade-offs.

The IVI is estimated through a hybrid method that considers the number of visible entities (e.g., objects, actions), retained interpretability weight, and visual clarity post-obfuscation. RII increases protection when re-identifiability risk reaches the threshold, while IVI ensures intelligibility is not unnecessarily degraded. This dual scoring enables detailed control over what is obfuscated. In addition, feature-level privacy directives (e.g., “always hide logos”) are consistently enforced by the ontology-driven reasoning engine and ensure that user-defined red lines override contextual inference when necessary.

To empirically illustrate the privacy–intelligibility trade-off on sensor-acquired video data, Table 6 presents example scenarios with varying RII and IVI scores, system-inferred privacy levels, and their corresponding obfuscation strategies. The Intelligibility Score represents the approximate proportion of semantic content preserved after obfuscation. It is computed using a weighted combination of IVI, the presence and visibility of key visual features (e.g., faces, actions, and objects), and their semantic weights, outlined in Table 3.

In cases where an unregistered individual is captured in a public setting, the framework detects soft features and calculates an RII score. If the RII score passes the threshold, the framework automatically applies obfuscation to the individual’s face and associated soft features. This ensures individuals without explicit consent are protected against re-identification risks. In another scenario, a registered user set a red line to “never show jacket”, and the framework enforced selective obfuscation, masking only the user’s jacket while keeping the rest of the face and body visible. Although the RII was moderate (0.4), this user-defined rule took precedence over contextual inference, validating the ability of the framework to enforce user autonomy through red lines.

Compared to prior efforts, such as Hasan, Shaffer, Crandall, and Kapadia [9], who achieved only 5% object masking accuracy using cartoonisation and reported a 95% identifiability rate among users, the proposed ontology-driven framework demonstrates a significant performance advantage. Across 7410 evaluated frames, it achieved 77.8% privacy protection accuracy in real-time video streams. Although 22.2% of users were still able to recognise at least one individual, this identifiability was mainly attributed to low-resolution constraints (224 × 224 px) used for real-time processing efficiency.

Furthermore, unlike static masking techniques that apply uniform filters across content, the proposed framework dynamically adjusts obfuscation based on entity sensitivity, user-defined privacy settings and red lines, soft biometric recognition, and RII and IVI trade-off scoring. This enables detailed, transparent, and explainable privacy protection aligned with the principles of Contextual Integrity, as well as the accountability and data minimisation requirements of the GDPR.

In conclusion, balancing intelligibility and re-identifiability requires more than just masking; it requires adaptive, context-aware enforcement that accounts for human perception, risk levels, and ethical protection. By combining RII–IVI analytics, ontology-based privacy reasoning, and user-driven preferences, the proposed framework offers a flexible, adaptive method to privacy in real-world multimedia settings.

### 4.2. Privacy Protection Effectiveness

The evaluations of proposed privacy-preserving methods included user studies and quantitative evaluations to measure their effectiveness on data protection, alongside user convenience and transparency. The ontology-driven privacy protection, supported by user-defined red lines, such as “always hide logos”, ensures that personal preferences are always respected, regardless of contextual inference or predicted privacy level (see Table 4). This ensures that individual privacy preferences are respected in all scenarios.

The results show that 77.8% of participants were unable to recognise any individuals within obfuscated videos, while 22.2% of participants identified at least one user, mainly because of false negatives from the face recognition module. The obfuscation techniques were rated “highly effective”, with 85.2% of participants rating them “very effective” and 14.8% rating them as “somewhat effective”. Regarding overall privacy protection, 74.1% “strongly agreed” and 22.2% “agreed” that the framework provided strong privacy protection. Users also expressed confidence in data handling, with 53.6% reporting “very confident” and 39.3% reporting “confident” in the framework protection mechanisms.

**Figure 2:**
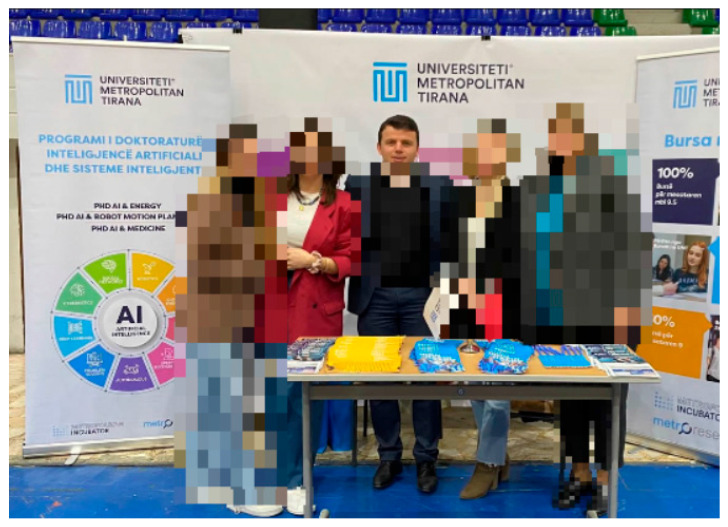
Dynamic and user-centric obfuscation, taking into consideration user preferences such as not wanting any general privacy but wanting to always hide the jacket.

A main challenge in privacy-preserving AI systems is balancing privacy with usability. Users evaluated the balance between privacy and intelligibility positively, with results showing that 71.4% of participants rated it “well balanced” and 28.6% rated it as “somewhat balanced”. Notably, 34% of participants who evaluated the framework stated that the video clarity suffered a reduction, particularly at higher privacy levels or when subjects appeared too close, as illustrated in Figure 2. These results reinforce the need for adaptive obfuscation methods that maintain intelligibility and ensure strong privacy protection. While prior works have reported anonymisation accuracy, they do not provide systematic metrics for soft biometric handling, contextual adaptability, or RII/IVI prediction. The proposed framework integrates and evaluates these aspects to address the identified gaps, and Table 7 compares different methods and the proposed framework in terms of privacy mechanisms and anonymisation accuracy.

The framework demonstrated its adaptability across multi-user scenarios, including users with and without predefined privacy preferences. For unregistered users, the Re-Identifiability Index (RII) was computed using soft biometric features such as hair colour, clothing, and gait. When the RII exceeded the risk threshold, the framework automatically applied obfuscation to the user’s face and soft biometric features, ensuring GDPR-compliant default privacy protection without any manual configuration. For registered users who specified red lines (e.g., “never show jacket”), the framework applied selective obfuscation only to the specified feature, in this case, the jacket, while leaving the rest of the frame unobscured. This showcases the detailed, user-respecting nature of the framework and its ability to distinguish between general privacy logic and user-enforced exceptions. This selective obfuscation results in minimal visual disruption, preserving full intelligibility of the user’s face and actions while respecting specific privacy directives.

This comparison highlights the robustness of the proposed framework and its ability to dynamically adapt privacy protection levels based on context, user-defined constraints, and re-identifiability risk. Unlike current privacy protection methods, our framework protects multiple dimensions of identity while maintaining intelligibility in most conditions.

To further demonstrate the robustness of the proposed framework, Table 8 provides a feature-level comparative evaluation against state-of-the-art privacy protection methods. Unlike current methods, which focus on single modalities or fixed obfuscation strategies, the proposed ontology-driven framework introduces dynamic adaptability, contextual reasoning, and risk-based handling of soft biometric features. This highlights the significant improvements introduced by our ontology-driven framework in achieving stronger privacy protection, retaining higher levels of scene intelligibility handling, and ensuring compliance with data protection principles.

In summary, the proposed framework advances current privacy-preserving methods by enabling real-time, context-aware, and user-specific privacy protection, validated through both quantitative results and comparative evaluation. Unlike earlier works such as Badii [11,12,13], which focused on static or semi-dynamic privacy filters with limited user control and no soft biometric modelling, the proposed ontology-driven framework introduces dynamic adaptation, user red line enforcement, soft biometric risk handling, and explainable reasoning. These improvements address gaps in current methods and demonstrate strong potential for GDPR-compliant deployment in real-world AI environments.

### 4.3. Computational Performance

The computational efficiency of the proposed framework was evaluated in terms of processing speed, inference time, and scalability across different privacy levels. Real-time performance testing was carried out on sensor-acquired video streams, evaluating running times on CPU and GPU-accelerated setups under different privacy setting conditions. The framework delivers real-time execution at 163 ms per frame under the Low Privacy setting. However, the use of stricter privacy settings, where multiple faces, objects, emotions, and actions need to be identified and obfuscated, increased the execution time to 735 ms per frame. GPU acceleration made operation processing more efficient because it minimised latency regardless of scene complexity.

For example, face recognition processing time was reduced from 440 ms on the CPU to 92.73 ms per frame on the GPU. Similarly, action recognition processing improved from 15,880 ms on the CPU to 193 ms on the GPU. Other modules benefited similarly, as summarised in Table 9.

Compared to current methods reported by Frome, Cheung, and Abdulkader [8], which highlight processing times of 7–10 s per image, indicating severe limits in applicability for real-time video processing. In comparison, the proposed framework shows a significant advantage in achieving low-latency, frame-level privacy protection suitable for real-time applications.

The privacy engine of the framework was evaluated for latency in decision-making and obfuscation. Steps such as RII computation, scene sensitivity classification, aggregation of privacy levels, and soft feature obfuscation were measured, with the total reasoning and obfuscation latency ranging between 1.4 and 5.8 ms per frame. Obfuscation methods show varying computational costs, with pixelation completed on average at 3.06 ms, blurring at 540.73 ms, and GAN-based anonymisation at 2138.65 ms. Such results highlight the trade-off between privacy strength and processing overhead (e.g., GANs offer the strongest anonymisation but incur the highest latency), summarised in Table 10.

To enhance efficiency, the framework implements a module-on-demand strategy, executing each recognition module individually on the GPU only when required. This design functions at the highest efficiency by avoiding unnecessary data processing and executing modules only when needed. For example, if no faces are detected, the face authentication and face obfuscation tasks are not used, and preserve resources. This design avoids the overhead identified with multiprocessing, which required 74,436.24 ms per frame, or threading that processed frames at 23,860.48 ms, while maintaining real-time feasibility.

Overall, the evaluation confirms that GPU acceleration, ontology-driven reasoning, and context-aware module execution are crucial for achieving real-time privacy protection without sacrificing accuracy. The framework maintains a balance between performance, adaptability, and privacy robustness, and at the same time, it remains scalable and efficient across a variety of contexts.

### 4.4. User Satisfaction and Usability

The evaluation of the framework’s usability was based on user surveys and direct interaction tests that measured usability, privacy assurance, and responsiveness. The participant sample was mainly composed of younger users, with 67.9% aged 18–24, 10.7% aged 25–34, 17.9% aged 35–44, and 3.6% made the 55+ age group. This demographic profile contributed to a higher familiarity with privacy protection tools such as face filters and other obfuscation features usually used in social media applications. As a result, participants demonstrated heightened expectations for achieving an optimal balance between obfuscation strength and scene intelligibility.

Survey results indicated broad confidence in the framework’s adaptability and effectiveness. According to 85.7% of participants, adaptable privacy controls improved the ability to control their data. Also, 88% of participants reported that applied protection was sufficiently maintained without overly compromising intelligibility. To enforce this, 85% affirmed the framework remained responsive, even under multi-user and dynamic privacy adaptations, and 92.9% showed trust in how well the framework protects sensitive data. However, 34% of participants stated that heavy obfuscation affected scene intelligibility, or when users were close to the camera. This shows that stronger privacy protection ensures confidentiality, but it can also weaken scene intelligibility.

**Figure 3:**
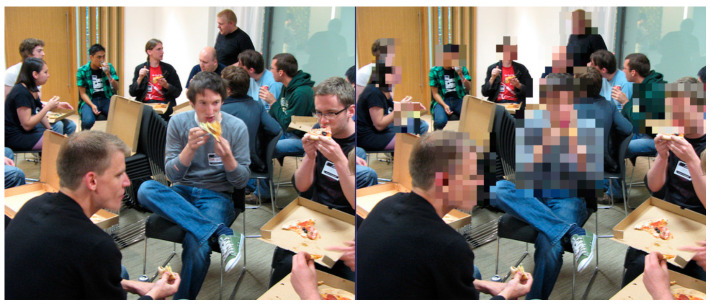
Sample image from the COCO dataset [38] with many individuals present, which demonstrates that the proposed obfuscation method effectively protects sensitive features without substantially degrading the overall video quality or scene intelligibility.

This reflects the central RII–IVI trade-off: as the RII increases, prompting stronger obfuscation, the IVI tends to decrease. This is particularly evident in multi-user or high-risk settings where full masking is applied to faces and soft biometric features. Nevertheless, the framework preserves intelligibility wherever possible by using targeted obfuscation and preserving unmasked content when privacy risks are low. Importantly, user-defined red lines, such as “always hide jacket” (Figure 2), were honoured in different scenarios. This enforcement of user-defined red lines, regardless of context, improved trust and demonstrated the loyalty of the ontology-based privacy engine. Figure 3 shows obfuscation techniques without impactful effects on video quality while preserving scene quality.

**Figure 4:**
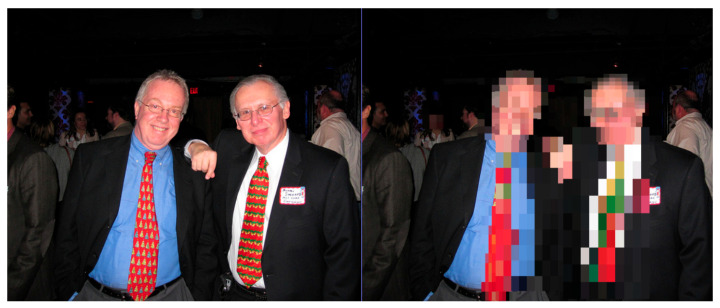
Example from the COCO dataset [38] illustrating how the obfuscation technique can reduce scene quality in complex multi-user scenarios.

In contrast, Figure 4 illustrates a case in which heavy obfuscation leads to notable reductions in scene clarity when multiple users are present in close proximity.

Transparency was another critical factor in user trust, where 71.4% of users agreed that the framework offered a good balance between privacy and usability, and 28.6% found the balance “somewhat” acceptable, pointing to a need for clearer explainability mechanisms. Users expressed interest in better explainability of how and why some privacy decisions are made, particularly when red lines or contextual obfuscations intersect.

### 4.5. Error Analysis and Framework Limitations

**Figure 5:**
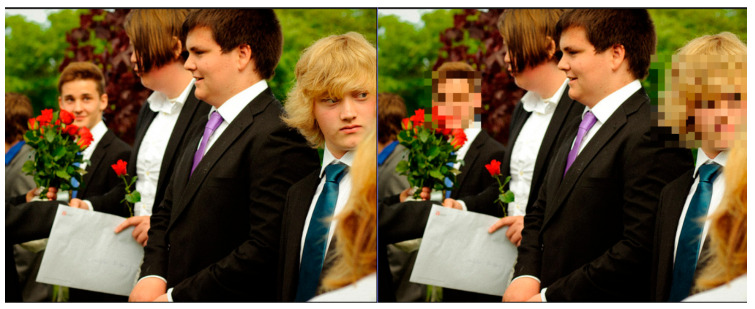
Example from the COCO dataset [38] showing a failure case in the face recognition module, where non-frontal face orientations were not detected, resulting in missed obfuscation.

While the proposed framework effectively enforces privacy protection, certain challenges and limitations were identified during the evaluation. One key limitation arises when face and object recogniser systems fail to identify targets specifically in low-light conditions, when objects are partially obscured, or in low-resolution video frames. In some instances, incorrect identification resulted in incomplete obfuscations, leading to potential risks. For example, face recognition failures occurred when users were partially visible due to occlusions or when image processing steps (e.g., resolution downscaling for efficiency) caused faces within sensor-acquired frames to become too small for reliable recognition of PII features. A clear deficiency occurred when human faces appeared in non-frontal orientations, which led to the failure of the face detection module and, as a result, missed obfuscations of faces, as shown in Figure 5.

In addition to facial recognition issues, limitations were observed in soft biometric recognition and RII-based privacy enforcement. In certain frame-level data instances, soft features such as hair colour, skin tone, or clothing logos were misclassified due to lighting variations or partial occlusion. This occasionally led to inflated RII scores and unnecessary obfuscation, reducing intelligibility without increasing actual privacy protection. Conversely, in other cases, weak recognition of soft features caused underestimated RII values, resulting in insufficient protection for potentially identifiable individuals. These findings suggest that confidence-aware soft biometric recognition and threshold calibration could improve both accuracy and interpretability in RII-driven privacy decisions.

Another limitation is the misclassification of entity sensitivity, such as classifying a semi-private environment as public areas, producing incorrect overall privacy settings. This issue was identified in indoor environments, such as an office area, where distinguishing between private and public contexts proved difficult and misleading. To improve such scene classification, the proposed framework integrates ontology rules and context interpretation that improve consistency.

Real-time performance was impacted as privacy settings increased, introducing higher computational overheads. While Low Privacy settings maintain an average processing time of 163 ms per frame, the use of High Privacy settings in densely populated scenes increased processing time to 735 ms per frame. This demonstrates the trade-off between privacy protection and framework performance when operating on hardware-limited devices.

Overall, while the proposed framework shows strong privacy protection capabilities, further improvements are needed in soft biometric handling, improved recognition accuracy, context misclassification, and hardware optimisation. Addressing these areas will further improve the adaptability and effectiveness of the proposed framework in real-world and privacy-sensitive environments.

### 4.6. Summary of Key Findings

The evaluation of the proposed privacy protection framework confirms its effectiveness in balancing privacy, usability, and computational efficiency. The adaptive framework combines ontology-driven reasoning, user-defined red lines, and contextual sensitivity analysis to dynamically adjust privacy levels while at the same time retaining semantic clarity. Key innovations, such as the Re-Identifiability Index (RII) and user-defined red lines such as “always hide logos” or “never show jacket”, enabled detailed, persistent privacy control, even across changing scenes and user contexts. These features proved critical in multi-user environments and when handling unregistered users, where privacy levels were inferred using soft biometrics and contextual risk.

Quantitative results demonstrated that privacy protection techniques, including pixelation, blurring, and GAN-based anonymisation, reduce the risk of subject re-identification. In 77.8% of cases, participants were unable to recognise individuals in obfuscated videos. Privacy enforcement was rated “highly effective” by 85.2% of participants, and 92.9% reported confidence in the ability of the framework to protect private information. Despite a 34% drop in perceived clarity under High Privacy settings, 71.4% of participants viewed the framework as achieving a “good balance” between privacy protection and scene intelligibility. These findings highlight the effectiveness of the proposed framework over current methods.

Compared to prior static methods [8,9,10,14,15,44], which offered static privacy enforcement with limited adaptability, the proposed framework achieves a 96.3% protection success rate while dynamically adapting to user-centric requirements, context-awareness, and real-time video processing. As shown in Table 9, the proposed framework outperforms current methods across multiple privacy protection dimensions, including context awareness, soft biometric handling, and intelligibility preservation, thereby confirming its practical viability. The framework also complies with GDPR principles through data minimisation, opt-out mechanisms for soft biometrics, and transparent user controls. These compliance requirements are legal obligations and also guide the design of the proposed framework in ensuring efficient and real-time processing without affecting privacy.

Performance evaluations confirmed that GPU acceleration enabled real-time processing, with acceptable execution times of 163 ms per frame under Low Privacy and 735 ms under High Privacy settings. Recognition module optimisations delivered up to 98.8% improvement in inference speed, making the framework scalable and suitable for real-time deployment.

Overall, findings establish that the proposed framework meets scalability and usability while being compliant with GDPR, demonstrating its potential for deployment in a variety of AI applications, including social media, surveillance, and smart environments. Limitations remain for low-resolution frame-level scenarios and complex scene classifications, which offer promising directions for future enhancement, including improving recognition reliability of PII features, expanding explainability, and intelligibility-privacy trade-off refinement, especially in low-resolution or high-risk scenarios.

## 5. Discussion

Current privacy-preserving AI models use fixed anonymisation rules that apply uniform privacy settings that do not take into consideration individual privacy preferences or contextual factors. These static anonymity techniques lead to primary issues such as overprotection that reduces intelligibility or under-protection, providing inadequate protection of sensitive information. As noted by Shu, Zheng, and Hui [7], current systems employ either manual intervention or predefined rules for privacy protection. Additionally, previous multimodal AI architectures, although combining multiple recognition functionalities, have been criticised by Rivadeneira, Silva, and Colomo-Palacios [16] for the absence of user control and transparency of the decision-making process. Similarly, Badii and Al-Obaidi [11,12] and Badii, Tiemann, and Thiemert [13] highlighted the importance of context-driven decision making in privacy protection AI. They also state that current models fail to account for situational variations in privacy sensitivity and the need for empowering users to define rules that reflect complex information needs.

In contrast, the proposed framework addresses the above limitations by introducing an ontology-driven and user-centric method that dynamically updates privacy settings based on user-defined privacy rules, the sensitivity of detected entities, contextual analysis, and real-time privacy level predictions. Unlike current methods [7,8,9,10,14,15], it integrates multimodal recognition (faces, objects, scenes, actions, emotions, and soft biometrics) with ontology-based reasoning to deliver context-sensitive and user-specific privacy enforcement. Other important advantages include enforcement of user-defined red lines (e.g., “always hide jacket”), the use of dual metrics including RII and IVI that balance privacy and intelligibility, and GPU-accelerated execution for real-time deployment. These design choices collectively ensure adaptive, explainable, and GDPR-compliant privacy protection and improve user trust and system scalability.

Experimental results demonstrate the framework’s privacy protection success rate of 96.3%, outperforming current methods such as that of Shan, Wenger, Zhang, and Li [44], which achieved 80% accuracy on static images, and which lack contextual and user-centric adaptation. Participants reported high levels of satisfaction, with 85.2% rating privacy protection as “highly effective”. Also, 71.4% stated that the framework achieved a good balance between privacy protection and scene intelligibility, and 28.6% stated that the balance was adequate. This shows that the proposed framework maintained a strong balance between privacy protection and scene intelligibility, highlighting the need for context-aware and personalised privacy controls in environments.

GPU acceleration proved essential in delivering real-time processing and reducing processing delays across recognition tasks (e.g., face recognition improved from 440 ms to 92.73 ms and scene recognition from 250 ms to 5.82 ms, Table 9). Such performance optimisations make the framework suitable for deployment in resource-constrained environments, and also address key limitations stated by Sezer, Dogdu, and Ozbayoglu [27], Wang and Deng [45], and Abadi, Chu, and Goodfellow [46].

Nonetheless, the framework has limitations. Recognition accuracy degrades under low-light, occlusion, or low-resolution conditions, which leads to occasional missed obfuscations. Scene misclassification in complex environments can result in incorrect privacy level predictions. Additionally, some obfuscation methods, such as GAN-based anonymisation, provide better scene intelligibility but introduce delays, by processing frames at 2138.65 ms. The use of lighter weight alternatives, such as pixelation, showed improvements by processing frames at 3.06 ms, hence enabling real-time privacy protection.

Moreover, while 96.3% of participants reported effective privacy adaptation, 34% stated difficulty understanding how their data was processed. This highlights the need for improved transparency and user feedback mechanisms. Future improvements should include explainability features and better visualisation of privacy decisions.

In scenarios where intelligibility is important, such as in workspaces, privacy protection must be considered for the Re-Identifiability Index (RII) and the Information Value Index (IVI) of visual elements. Formalising these dual indicators and integrating rule-based reasoning with machine learning could support more intelligent obfuscation decisions that protect privacy without compromising scene intelligibility.

Overall, results show that the proposed framework improves adaptive privacy protection by combining user-centric privacy settings, ontology-driven reasoning, and multi-modal recognition to achieve a better balance between privacy protection and scene intelligibility. The integration of user-defined rules, entity and contextual sensitivity, and the Re-Identifiability Index achieves adaptive and GDPR-compliant privacy protection that is scalable and explainable. These results highlight that effective privacy protection requires user-centric and privacy rules that can adapt to scene contexts and sensitivity. By applying privacy protection to data captured by visual sensors, the framework demonstrates that it is applicable and effective in real-world, vision-based systems.

## 6. Conclusions and Future Work

The proposed framework introduces a user-centric, ontology-driven privacy protection architecture that adaptively changes privacy settings responsive to the user preferences, privacy-context sensitivity, and multimodal recognition of entities in vision-sensor data streams. It performs real-time recognition of faces, objects, scenes, emotions, actions, and soft biometrics, combined with an ontology-based reasoning engine that considers user preferences and contextual privacy sensitivity to optimise the balance of privacy protection and scene intelligibility. In contrast to current static models, the proposed framework provides detailed, real-time, and entity-specific obfuscation guided by the Re-Identifiability Index (RII), and user-defined red lines, enabling a transparent, explainable, and GDPR-compliant privacy adaptation.

Experimental evaluation demonstrated a 96.3% privacy protection success rate (Table 9 and Table 10) and maintained an optimal balance between privacy and intelligibility, maintaining scene intelligibility above 60% under high privacy settings (Table 8). GPU acceleration and optimised execution pipelines reduced inference times significantly (e.g., face recognition from 440 ms to 92.73 ms, scene recognition from 250 ms to 5.82 ms) and kept privacy engine decision-making and obfuscation latency within real-time thresholds (Table 10). User feedback confirmed the effectiveness of the framework, with 85.2% of participants rating privacy protection as “highly effective” and 71.4% reporting a good balance between privacy and intelligibility (Table 7). These results highlight the advantages of the framework over current methods by combining real-time performance, contextual adaptability, multimodal integration, and user-centric privacy protection.

Nonetheless, challenges remain. Recognition accuracy degrades under low-light, occlusion, or low-resolution conditions, leading to occasional misclassifications and excessive fallback protections. GAN-based anonymisation, though effective, introduces prohibitive latency (2138.65 ms per frame), limiting its suitability for real-time use. Addressing these challenges is necessary for improving robustness in complex environments.

Future research should focus on improving recognition accuracy in challenging conditions to reduce misclassifications of entities in sensor-acquired frames and the fine-tuning of the context-aware classification ontology. Privacy-oriented, light-weight gait and soft feature recognition, and detailed testing with larger and more diverse datasets will contribute to better robustness and generalisability. Additionally, integrating Explainable AI (XAI) [47] mechanisms into multimodal AI frameworks will enable users to have a better understanding of the scene contexts for the context-specific privacy safeguards implemented within this architecture, hence increasing transparency and user trust.

In conclusion, the proposed framework advances the field of privacy-preserving AI by combining ontology-driven reasoning, multimodal recognition, dual metrics (RII/IVI), user-defined rules, and real-time execution to deliver dynamic, context-aware privacy protection. Unlike current methods, it adapts dynamically to user preferences, contextual sensitivity, and re-identification risks, which ensures that privacy safeguards are effective and explainable. By integrating transparency, usability, and strong privacy guarantees, this research provides a foundation for next-generation AI systems that are required to operate responsibly and transparently in sensor-based, real-world environments.

## Figures and Tables

**Table 1:** Soft biometric features and re-identifiability weights.

Feature	Weight (±Variance)	Source
Gait	0.8–0.98 accuracy	Bari and Gavrilova [18]
Hair Colour	3.1 ± 0.7	Corbishley, Nixon, and Carter [19]
Gender	2.1 ± 0.6	Corbishley, Nixon, and Carter [19]
Hair Type	2.0 ± 0.6	Corbishley, Nixon, and Carter [19]
Skin Colour	1.6 ± 0.4	Corbishley, Nixon, and Carter [19]
Age	0.5 ± 0.8	Corbishley, Nixon, and Carter [19]
Clothing Style	Contextual	Moctezuma, Conde, Diego, and Cabello [32]

**Table 2:** Privacy enforcement rules based on ontology and user-defined red lines.

User Privacy Setting	Scene Sensitivity	Object Sensitivity	Action Sensitivity	Emotion Sensitivity	Re-Identification Features	Resulting Privacy Level	Enforcement Action
No Privacy	Non-sensitive	Non-sensitive	Non-sensitive	Non-sensitive	Any	No Privacy	No obfuscation
Low Privacy	Sensitive	Sensitive	Sensitive	Sensitive	Any	Low Privacy	Obfuscate sensitive elements only (scene, object, action, emotion)
Medium Privacy	Semi-sensitive or Sensitive	Semi-sensitive or Sensitive	Semi-sensitive or Sensitive	Semi-sensitive or Sensitive	Any	Medium Privacy	Obfuscate all semi-sensitive and sensitive entities
High Privacy	Any	Any	Any	Any	Any	High Privacy	Full obfuscation, including faces, soft biometrics, actions, objects
Auto Privacy	Any	Any	Any	Any	Any	Predicted	Adaptive obfuscation based on historical patterns, context, and RII score
User-defined red lines (objects, logos, users)	Any	Any	Any	Any	Any	User Specified	Always obfuscate specified features, objects, or individuals regardless of context or predicted privacy.
Any	Any	Any	Any	Any	RII exceeds threshold	Soft feature obfuscation	Obfuscate high-risk soft features (e.g., hair, clothing) selectively based on RII

**Table 3:** IVI evaluation for a single frame with retained interpretability after obfuscation of RII features.

Feature	RII (Re-Identifiability)	Interpretability Weight	Obfuscated?	IVI Contribution
Face	0.95	0.45	Yes	0.0
Gait	0.8	0.3	Yes	0.0
Hair Type	0.02	0.03	No	0.03
Hair Colour	0.03	0.02	No	0.02
Age	0.08	0.05	No	0.05
Gender	0.03	0.25	No	0.25
Bag	0.01	0.1	No	0.1
Bag colour	0.012	0.03	No	0.03
Bag logo	0.022	0.02	No	0.02
Dress	0.1	0.15	No	0.15
Dress colour	0.015	0.05	No	0.05
Dress logo	0.023	0.03	No	0.03
Hat	0.1	0.2	No	0.2
Hat colour	0.014	0.03	No	0.03
Hat logo	0.02	0.02	No	0.02
Jacket	0.1	0.15	No	0.15
Jacket colour	0.015	0.03	No	0.03
Jacket logo	0.024	0.02	No	0.02
Pants	0.11	0.3	No	0.3
Pants colour	0.02	0.03	No	0.03
Pants logo	0.03	0.02	No	0.02
Shirt	0.01	0.1	No	0.1
Shirt colour	0.015	0.03	No	0.03
Shirt logo	0.023	0.02	No	0.02
Shoes	0.02	0.1	No	0.1
Shoes colour	0.01	0.03	No	0.03
Shoes logo	0.02	0.02	No	0.02
Shorts	0.1	0.1	No	0.1
Shorts colour	0.015	0.03	No	0.03
Shorts logo	0.02	0.02	No	0.02
Skin tone	0.02	0.02	No	0.02
Skirt	0.1	0.1	No	0.1
Skirt colour	0.015	0.03	No	0.03
Skirt logo	0.02	0.02	No	0.02
Sunglasses	0.012	0.1	No	0.1
COCO Object Types (e.g., person, vehicle, furniture, electronics)	0.01	0.1–0.5	No	0.1–0.5
Combined Soft Biometrics (Face + Hair + Jacket)	0.91 normalised	0.63	Yes (only jacket)	0.76

**Table 4:** Framework parameters and thresholds for recognition modules, privacy decision-making, and obfuscation methods used in the experimental evaluation.

Component	Model	Dataset	Parameters Used
Face Recognition	MTCNN	VGGFace2 [37] consists of nine thousand identities with 80 to 800 images for each identity, and 3 M+ images in total.	Confidence threshold: 0.7
Object Recognition	YOLOv5s	COCO [38] consists of more than 200 K labelled images and 80 object categories.	Confidence threshold: 0.25
Scene Recognition	AlexNet	Places365 [39] consists of 1,803,460 images with 3068 to 5000 images per class and labelled across 365 scene categories.	Input resolution: 224 × 224
Action Recognition	SlowFast R50	Kinetics [40] consists of 650 K videos from 700 human action classes, in a wide range of activities.	Batch size: 32 frames, sampling rate: 2
Emotion Recognition	EfficientNet	FER2013 [41] consists of 35,887 grayscale images of faces, annotated across 7 emotion categories (angry, disgust, fear, happy, sad, surprise, neutral).	Input resolution: 48 × 48, confidence threshold: 0.6
Privacy Thresholds	RII/IVI	-	RII obfuscation triggered at >0.1 and IVI maintained >0.4
Obfuscation Methods	Pixelation	-	Pixelation block: 10 px
Auto Privacy	Random Forest	Trained on users’ historical data	Trained on historical interactions and contextual features
Soft Feature Recognition	YOLO trained on bespoke dataset	Colorful Fashion Dataset [42], bespoke for clothing dataset (10 classes)	Number of classes: 10, confidence: 0.4
Logo Detection	YOLO trained on bespoke dataset	Colorful Fashion Dataset [42], bespoke for logo dataset (22 classes)	Number of classes: 22, confidence: 0.4

**Table 5:** Balance between information value and privacy sensitivity for multimodal features used in the framework.

Feature	Information Value (Usability)	Privacy Sensitivity (Re-Identifiability Risk)	Comment
Face	High	Very High	Critical for identification
Gait	Medium	High	Useful for action recognition
Hair Type	Low-Medium	Medium	Somewhat distinctive, minor contribution to scene understanding
Hair Colour	Low	Medium	Minor scene value, moderate privacy risk
Clothing Style	High	Medium	Could reveal user profile
Object Carried (e.g., Bag)	Medium	Low	Provides some scene context, minimal risk unless branded
Emotion (Face Expression)	Medium	High	Important for interaction value, but reveals sensitive emotional states
Background Scene (Park, Home)	High	Low	High usability for context, low risk unless it contains private information

**Table 6:** Privacy–intelligibility trade-offs across representative frame contexts.

Frame Context	RII	IVI	Final Privacy Level	Obfuscation Applied	Intelligibility Score
Public Park—Social Media	0.7	0.4	High	Face and gait masked	60%
Office	0.2	0.8	Low	Only the sensitive object is masked	85%
Home—Family Gathering	0.5	0.6	Medium	Face, soft features, sensitive objects	70%
Classroom—Multi-user	0.4	0.6	Medium	Faces, selective objects, logo masking	72%
Public Square—Unknown user	0.8	0.3	High	Face + clothing + gait obfuscated	55%

**Table 7:** Comparative performance of privacy protection methods.

Method	Privacy Mechanism	Anonymisation Accuracy
Frome and Cheung [8]	Blurring (face and number plates)	93.6% (metrics on number plates only)
Hasan and Shaffer [9]	Cartoonisation	5% (object masking)
Ren, Lee, and Ryoo [10]	Image modification	80.25% (on static faces)
Badii, Tiemann, and Thiemert [12]	Blurring	42.80% (on static images)
Zhou, Pun, and Tong [14]	Face pixelation	60% (face only)
Sweeney and Malin [15]	k-Same pixel	71% anonymised faces
Proposed Framework	Ontology + RII + Obfuscation	77.8%

**Table 8:** Feature-level comparison of static, semi-dynamic, and context-aware privacy protection methods with the proposed ontology-driven framework.

Feature/Aspect	Static Data Protection [8,9,10,14,15]	Partially Dynamic Protection [7]	Context-Aware Privacy Filters [11,12,13]	Proposed Ontology-Driven Privacy Framework
Context-Awareness	No	Partial (fixed rules)	Medium (scene elements)	Full (context, user settings)
Adaptability to User Preferences	No	Limited (static settings)	No	High (dynamic + user-defined red lines)
Privacy Adaptation (Sensitivity)	Low	Medium	Medium (face, skin, and body)	High (hierarchical, context-driven)
Intelligibility Preservation	Poor	Medium	High (pleasantness and intelligibility)	High (selective obfuscation)
Soft Biometric Handling Re-Identification Risk	No	No	No	Yes (Gait, Hair, Clothing, etc.)
Auto Privacy Prediction	No	No	No	Yes (Random Forest prediction)
GDPR Compliance	No	Partial	Not explicitly addressed	Strong (adaptive + user control)
Real-time Performance	Limited (still images)	Moderate (basic rule engines)	Partial (MediaEval real-time filters)	High (GPU-accelerated, real-time video)
Overall User Satisfaction	N/A	N/A	Subjective evaluation on pleasantness only	88% positive, 85% acceptable clarity

**Table 9:** GPU acceleration impact on recognition modules for real-time privacy protection.

Model	Baseline Execution Time (CPU)	Optimised Execution Time (GPU)	Improvement (%)
YOLOv5 (Object Recognition)	150 ms	17.62 ms	88.3% Faster
MTCNN (Face Recognition)	440 ms	92.73 ms	78.9% Faster
SlowFast R50 (Action Recognition)	15,880 ms	193 ms	98.8% Faster
AlexNet (Scene Recognition)	250 ms	5.82 ms	97.7% Faster
Emotion Recognition	19 ms	8.31 ms	56.3% Faster

**Table 10:** Privacy engine decision-making and obfuscation latency per frame.

Step	Description	Average Time (ms)
RII computation	Compute the Re-Identifiability Index from soft biometrics	1.0–3.0 ms
Scene sensitivity classification	Determine the highest scene privacy level	0.02–0.2 ms
Highest privacy aggregation	Combine privacy levels from users, scene, emotion, action	0.02–0.1 ms
Soft feature obfuscation	Pixelate features (logo, clothes, gait, hair, accessory)	0.3–2.5 ms
Total decision + obfuscation latency	Privacy engine reasoning + obfuscation application	1.4–5.8 ms

## Data Availability

The raw data supporting the conclusions of this article will be made available by the authors on request.

## Appendix A

**Table A1:** Comparison of face recognition models.

Face Recognition Model	Accuracy (%)	Speed Frames per Second (fps)
FaceNet [48]	99.63%	20–30 fps
DeepFace [49]	97.35%	20–25 fps
ResNet [50]	99.60%	20–30 fps
VGGFace [51]	98.95%	15–20 fps
ArcFace [52]	99.83%	20–30 fps
The Histogram of Oriented Gradients (HOG) detector [53]	85–90%	30–60 fps
Multi-task Cascaded Convolutional Networks (MTCNN) [54]	94.4%	16–99 fps

**Table A2:** Comparison of scene recognition models.

Architecture	Model	Accuracy	Speed (fps)
Convolutional Neural Networks (CNNs)	ResNet-50 [50]	76% accuracy on ImageNet	Approximately 100 fps
	ResNet-101 [50]	Around 77% accuracy on ImageNet	Approximately 60 fps
	ResNet-152 [50]	Around 78% accuracy on ImageNet	Approximately 40 fps
	VGG16 [55]	Around 71.5% accuracy on ImageNet	Approximately 40 fps
	VGG19 [55]	Around 71.9% accuracy on ImageNet	Approximately 30 fps
	AlexNet [56]	Around 85% accuracy on ImageNet	Approximately 205 fps [56]
Region-Based Convolutional Neural Networks (R-CNNs)	Faster R-CNN [57]	Around 73.2% accuracy on specific recognition tasks	Approximately 5 fps on an NVIDIA V100 GPU
	Mask R-CNN [58]	Similarly to Faster R-CNN for scene recognition tasks, around 75–80%	Approximately 2–5 fps on an NVIDIA V100 GPU
	Long-Short Term Memory (LSTM) [59]	Better accuracy than most models, at 85%	It comes with overheads as it uses recurrent networks and can process around 15 fps

**Table A3:** Comparison of object recognition models.

Architecture	Model	Accuracy	Speed Frames per Second (FPS)
Single-stage detectors	You Only Look Once (YOLO) [53]	63.4%	Approximately 45 FPS
	You Only Look Once (YOLOv5)	50%	Approximately 140 FPS
	You Only Look Once (YOLOv8/YOLOv10)	50%	30–60 FPS on a high-end GPU
	Single Shot Multibox Detector (SSD) [60]	76.9%	Achieves around 22 FPS
	RetinaNet [61]	39.6%	Approximately 8 FPS
	CenterNet [62]	47%	270 ms per frame or 3.7 FPS
	EfficientDet [63]	52.2%	Approximately 4 FPS
Two-Stage Detectors	CNN [57], AlexNet [56]	62–83%	Approximately 1 FPS
	R-CNN [50]	53.7%	At best 0.5 FPS
	Fast R-CNN [55,64]	68.8%	Achieves 6.67 FPS
	Faster R-CNN [57,60]	73.2%	5–10 FPS on high-end GPUs
	Spatial Pyramid Pooling Network (SPP-Net) [50]	59.2%	Achieves 2 FPS
	R-FCN [65]	76.6%	Achieves 5.88 FPS
	Mask R-CNN [58]	36.7%	Achieves 5.13 FPS

**Table A4:** Comparison of action recognition models.

**Model**	**Accuracy**	**Speed (FPS)**
CNNs [66,67]	97% on UCF101 dataset	Approximately 10–15 FPS
RNNs [68]	90% on HMDB51 dataset	5–8 FPS, slower due to the sequential nature
Two-Stream CNNs [68]	85% on UCF101 dataset	Approximately 7–10 FPS
Long-Short Term Memory [69]	75–80% on UCF101 dataset	10–15 FPS depending on the complexity and length of the video sequences
Temporal Segment Networks [70]	94% on UCF101 dataset	Approximately 20–25 FPS
Inflated 3D ConvNets (I3D) [71]	95% on Kinetics dataset	Approximately 10–15 FPS
SlowFast Networks [72]	96% on Kinetics dataset	Approximately 30–60 FPS

**Table A5:** Comparison of emotion recognition models.

**Model**	**Accuracy**	**Training Time**	**Computational Resources**
AlexNet [56]	84.7%	Moderate	Moderate (30 FPS on CPU or 300 FPS on GPU)
VGGNet [55]	92%	Long	High
ResNet [50]	96.43%	Long	Very High (110, 1202 layers)
RNNs [73,74]	84.8%	Long	Moderate
Faster R-CNN [57]	78.8%	Long	High
EfficientNet [41,55]	84.6%	Moderate	Moderate (faster than other models around 155FPS)
