# Correction: Topalli, N.; Badii, A. A User-Centric Context-Aware Framework for Real-Time Optimisation of Multimedia Data Privacy Protection, and Information Retention Within Multimodal AI Systems. *Sensors* 2025, *25*, 6105

**DOI:** 10.3390/s25226957

**Published:** 2025-11-14

**Authors:** Ndricim Topalli, Atta Badii

**Affiliations:** Department of Computer Science, University of Reading, Reading RG6 6AH, UK

In the original publication [1], there was a mistake in the caption for Figure 1. The correct caption appears below.

Figure 1. Framework architecture flowchart illustrating the process from recognition to ontology-driven privacy adaptation.

Atta Badii was not included in methodology as an author in the original publication. The corrected Author Contributions statement appears here.

**Author Contributions:** Conceptualization, N.T. and A.B.; methodology, A.B. and N.T.; software, N.T.; validation, N.T. and A.B.; formal analysis, N.T.; investigation, N.T.; resources, A.B.; data curation, N.T.; writing—original draft preparation, N.T.; writing—review and editing, N.T. and A.B.; visualization, N.T.; supervision, A.B.; project administration, A.B.; funding acquisition, N.T. and A.B. All authors have read and agreed to the published version of the manuscript.

There was an error in the original publication. The contextual linkage to the prior foundational works [30,31] had not been included.

A correction has been made to 2. Related Work, 2.2. Context-Aware and Multi-User Privacy Adaptation Techniques, after paragraph 3 and before paragraph 4:

The approach proposed in this research study is underpinned by earlier advances towards context-specific user-led requirements conflict resolution and prioritisation as provided by the UI-REF ontologically based methodology [30] and its application to context-specific privacy protection in multimedia data frames [31].

A correction has been made to 3.4. Person Re-Identification, paragraph 1, third sentence, to include one missed reference:

As Dantcheva, Elia, and Ross [37] highlight, a single soft biometric trait would not be unique enough to identify a subject, but their combination can significantly increase the probability of identity inference [34,38,39].

The following new references have been added:

30. Badii, A.; Fuschi, D.; Khan, A.; Adetoye, A. Accessibility-by-Design: A Framework for Delivery-Context-Aware Personalised Media Content Re-purposing. In *HCI and Usability for e-Inclusion, USAB 2009*; Lecture Notes in Computer Science; Holzinger, A., Miesenberger, K., Eds.; Springer: Berlin/Heidelberg, Germany, 2009; Volume 5889. https://doi.org/10.1007/978-3-642-10308-7_14.

31. Badii, A.; Einig, M.; Tiemann, M.; Thiemert, D.; Lallah, C. Visual context identification for privacy-respecting video analytics. In Proceedings of the IEEE 14th International Workshop on Multimedia Signal Processing (MMSP 2012), Banff, Canada, 17–19 September 2012; pp. 366–371.

39. Hoo, W.L.; Miron, A.; Badii, A.; Chan, C.S. Skin-based Privacy Filter for Surveillance Systems. In Proceedings of the 2015 International Conference on Systems, Signals and Image Processing (IWSSIP), London, UK, 10–12 September 2015; pp. 269–272. https://doi.org/10.1109/IWSSIP.2015.7314228.

With this correction, the order of some references has been adjusted accordingly.

The authors state that the scientific conclusions are unaffected. This correction was approved by the Academic Editor. The original publication has also been updated.
